# The MazEF Toxin-Antitoxin System Alters the β-Lactam Susceptibility of *Staphylococcus aureus*

**DOI:** 10.1371/journal.pone.0126118

**Published:** 2015-05-12

**Authors:** Christopher F. Schuster, Lukas Mechler, Nicoletta Nolle, Bernhard Krismer, Marc-Eric Zelder, Friedrich Götz, Ralph Bertram

**Affiliations:** 1 Department of Microbial Genetics, Faculty of Science, Interfaculty Institute of Microbiology and Infection Medicine Tübingen (IMIT), University of Tübingen, Tübingen, Germany; 2 Cellular and Molecular Microbiology, IMIT, University of Tübingen, German Center for Infection Research (DZIF), partner site Tübingen, Germany; University of Manchester, UNITED KINGDOM

## Abstract

Toxin-antitoxin (TA) systems are genetic elements of prokaryotes which encode a stable toxin and an unstable antitoxin that can counteract toxicity. TA systems residing on plasmids are often involved in episomal maintenance whereas those on chromosomes can have multiple functions. The opportunistic pathogen *Staphylococcus aureus* possesses at least four different families of TA systems but their physiological roles are elusive. The chromosomal *mazEF* system encodes the RNase toxin MazF and the antitoxin MazE. In the light of ambiguity regarding the cleavage activity, we here verify that MazF specifically targets UACAU sequences in *S*. *aureus in vivo*. In a native strain background and under non-stress conditions, cleavage was observed in the absence or presence of *mazE*. Transcripts of *spa* (staphylococcal protein A) and *rsbW* (anti-σ^B^ factor) were cut, but translational reporter fusions indicated that protein levels of the encoded products were unaffected. Despite a comparable growth rate as the wild-type, an *S*. *aureus mazEF* deletion mutant was more susceptible to β-lactam antibiotics, which suggests that further genes, putatively involved in the antibiotic stress response or cell wall synthesis or turnover, are controlled by this TA system.

## Introduction

*Staphylococcus aureus* is a Gram positive, opportunistic pathogen that is associated with many diseases, such as endocarditis, skin infections and the toxic shock syndrome [1]. From a clinical point of view, *S*. *aureus* is highly relevant due to the increasing prevalence of methicillin/multi-drug resistant strains in hospitals and more recently also communal areas [2,3]. In addition, antibiotic treatment of *S*. *aureus* infections is complicated by small colony variants (SCV) [4] and persisters that reside in a drug-tolerant state. Persisters form a subpopulation of isogenic cells that is much less sensitive to antibiotics than the bulk of a culture [5]. The switch from the SCV or the persister state to normal growth is critical for relapsing infections, often encountered with *S*. *aureus* [6,7]. TA systems have been implicated in persister cell formation, as shown in a number of bacterial pathogens [8–10].

TA systems are small, commonly bicistronic genetic elements that are widespread throughout the prokaryotes and are encoded on plasmids or chromosomes. They consist of a stable toxic protein that impairs or kills the bacterium itself and an unstable antitoxin which counteracts toxicity [reviewed in 11]. Further proposed and validated functions of TA systems include plasmid maintenance, stress regulation and adaptation, growth control and programmed cell death [12–17]. Unraveling the functions of TA systems is challenging, as deletions of single loci often fail to generate apparent phenotypes [18].

Depending on the mode of action and type of the antitoxin, TA systems can be organized into at least six different types [11,19], from which only types I (RNA antitoxin) and II (protein antitoxin) have been identified in staphylococci to date [20–29]. In this genus, two different type II TA system families have been experimentally verified, namely *yefM/yoeB* (also called *axe/txe*) and *mazEF*. The *yefM/yoeB* system [30] encodes a ribosome dependent RNase and is present in at least two independent paralogues in *S*. *aureus* and *S*. *equorum* [22,23].

*mazEF* encodes the RNase MazF and the cognate antitoxin MazE and is one of the most intensively studied TA systems. Originally, the sequence specificity of *S*. *aureus* MazF was reported to be VUUV’ (V = A, C or G, where V may or may not be identical to V’) [24]. Later studies in *Escherichia coli* however, proposed UACAU as the main cleavage site, which had not been surveyed in the first publication [28,29]. Further experiments suggested that MazF cuts the *spa*, *sigB* and *hla* mRNAs *in vivo* in *S*. *aureus*, mainly based upon the observation that the abundance of these RNAs was reduced under the conditions tested [26].

In staphylococci, *mazEF* is located immediately upstream of the *sigB* locus, encoding the alternative sigma factor σ^B^ and the adjacent regulatory genes *rsbUVW* [25,31–33]. Due to several promoters and a weak transcriptional terminator, one short transcript containing only *mazEF* and several, larger transcripts comprising *mazEF*, *rsbUVW* and/or the *sigB* gene are produced [25,32]. A recently discovered *mazEF* orthologue termed *pemIK* differs from *mazEF* by localization on a plasmid and being unlinked to the *sigB* locus [34].

We here revisited *S*. *aureus mazEF* in order to disentangle the ambiguity regarding the *in vivo* cleavage specificity of MazF and to shed new light on the function of this TA system. MazF was found to cut *spa* and *rsbW* specifically at UACAU sites. However, as indicated by translational reporter fusions, expression of the encoded proteins appeared unaffected by MazF cleavage, which suggests only minor contributions to expression regulation of these genes. Notably, a markerless *mazEF* deletion strain was more susceptible towards penicillin, reflected by a decrease in the minimal inhibitory concentration (MIC) and enhanced killing in liquid cultures containing penicillin or oxacillin. Our findings indicate that *mazEF* controls further, as yet unidentified genes and thus contributes to regulating staphylococcal physiology.

## Materials and Methods

### Bioinformatical work

To create the synteny plot, a BLASTN of *sigB* from *S*. *aureus* NCTC 8325 (SAOUHSC_02298) against the NCBI *non-redundant (nr)* database limited to staphylococci (Taxid: 1279) with default values was performed. Then, a taxonomy tree was generated and all staphylococcal species with more than 50% coverage of the *sigB* gene were included in the analysis. The *mazE* gene annotation was inserted by hand into the sequences of *S*. *aureus* Newman and *S*. *pasteuri* SP1. To create the HG003 sequence, the *S*. *aureus* NCTC8325 data set was used and the *rsbU* gene repaired by inserting 11 bp from *S*. *aureus* Newman. The sequence of *S*. *equorum* SE3 was received by R. Rosenstein, University of Tübingen (personal communication). The gene bank files were then analyzed by EasyFig with BLASTN and default values (min len: 0, max e: 0.001, min ident value: 0) and shaded according to their identity.

The *spa* and *coa* transcript regions were aligned in Jalview using ClustalO and default settings.

### Bacterial strains and growth conditions

All strains, plasmids and oligonucleotides used in this study are given in S1 Table. *E*. *coli* and *S*. *aureus* cultures were generally grown in 10 ml liquid BM rich medium (1% (w/v) soy peptone, 0.5% (w/v) yeast extract, 0.5% (w/v) NaCl, 0.1% (w/v) K_2_HPO_4_ 3H_2_O, 0.1% (w/v) glucose), shaking at 37°C and 150 rpm in 100 ml baffled flasks, or on solid BM medium (with 1.5% (w/v) agar-agar) at 37°C unless stated otherwise. Knockout candidates were grown at different temperatures depending on the step in the recombination process. MHB (Mueller-Hinton-Broth) and BHI (Brain heat infusion) media were prepared according to the manufacturer’s recommendations and TSB (Tryptic soy broth) contained 1.7% (w/v) casein peptone (pancreatic digest), 0.3% (w/v) soy peptone, 0.5% (w/v) NaCl, 0.25% (w/v) K_2_HPO_4_ 3H_2_O. After autoclaving, 0.25% (w/v) sterile glucose was added.

### Molecular cloning

Gene annotations are according to *S*. *aureus* NCTC8325, which is largely isogenic to HG003 [35]. Genomic DNA was isolated as described previously [36]. Briefly, cells were lysed with lysostaphin and genomic DNA extracted via chloroform-isopentanol (1:24), precipitated in ethanol and rehydrated in water. Plasmids were isolated using the QIAGEN mini preparation kit according to the manufacturer’s protocol. For *S*. *aureus* cells, the protocol was adjusted as follows: the pellet of 6 ml overnight culture was used, the resuspended pellet was incubated 40–60 min with 15 μg of lysostaphin and buffer volumes of P1, P2 and P3 were doubled. PCRs were performed using Reprofast Polymerase and *S*. *aureus* HG003 genomic DNA or appropriate plasmids as templates. For conventional cloning, PCR products and plasmids were double digested with restriction enzymes in the appropriate buffer, heat inactivated when possible, and purified using a QIAGEN PCR purification kit. Ligations were done at 16°C in a thermo-cycler, using T4 Ligase and 1×ligase buffer and used to transform *E*. *coli* DH5α cells.

Cloning of individual constructs is described in the strains and plasmids list (S1 Table). For Gibson assembly, plasmid backbones were prepared by restriction digests, whereas inserts were always PCR amplified with Q5 polymerase. Assembly reactions were done as described by Gibson [37]. In short, 15 μl of assembly mix was incubated with 5 μl of DNA mix (100–200 ng of plasmid and 2.5×molar excess of inserts each) and incubated in a thermocycler for 1 hour at 50°C. Finally 10 μl of assembled fragments were used to transform *E*. *coli* DH5α cells. A double primer directed approach was exploited to introduce insertions, deletions and base exchanges into plasmids of interest,[38] using the primers listed in S1 Table. *E*. *coli* was made chemically competent by RbCl treatment [39] and transformed by heat shock. Competent *S*. *aureus* cells were prepared and electroporated according to standard protocols [40] with some alterations. Briefly, cells were grown in 100 ml BM medium to an OD_578_ of 0.5, washed three times with ice cold 10% glycerol, resuspended in 400 μl 10% glycerol and then stored in 75 μl aliquots at -80°C. For transformations, about 5 μg of plasmid DNA was added to the cells and incubated for 10 min at room temperature. The cells were then subjected to electroporation in a 2 mm electroporation cuvette (200 Ω, 25 μF, 2.0 kV), suspended in 950 μl BM, incubated shaking at 37°C or 30°C for at least 90 min and plated on selective BM agar.

### Chromosomal modifications

The *mazEF* knockout plasmid was constructed in such a way that the P_*mazEF*_ promoter and the *rho* independent transcriptional terminator downstream of *mazEF* [25] were left unaffected while replacing the *mazE* and *mazF* open reading frames with a *lox* flanked erythromycin resistance cassette. The knockout procedure was performed as previously described [41] and the resistance cassette removed [42] to yield a markerless *S*. *aureus* HG003 *mazEF* deletion mutant (ΔSAOUHSC_02303-SAOUHSC_02304).

### Sample preparation for primer extensions

10 ml cultures in 100 ml flasks were grown overnight in BM medium at 37°C and 150 rpm. Then these cultures were used to inoculate 50 ml fresh BM medium to an OD_578_ of 0.07 in 250 ml flasks and incubated for 3–3.5 hours at same conditions as above. Whenever necessary the medium was supplemented with chloramphenicol (10 μg/μl) to select for pRAB11-derived plasmids. Depending on the OD_578_, 15–50 ml of culture was harvested by 10 min centrifugation at 4,500×*g* and 4°C, and stored at -20°C until further processing. All experiments were done at least in duplicates. Changes to the protocol are indicated in the passages below. In case of HG003 and HG003Δ*mazEF*, 25 ml was harvested when each culture reached an OD_578_ of approximately 3.5 (about 3.25–3.6 hours). Parts of the cultures of HG003 and HG003Δ*mazEF* containing pRAB11-*spa-gpmCherry* were induced with 0.4 μM anhydrotetracycline (ATc) after 1.5 hours and 50 ml of all cultures were harvested after incubation for another hour.

HG003Δ*mazEF* cells containing either pRAB11-Pr-*mazEF*, pRAB11-Pr-*mazEF mazE*stop, pRAB11-Pr-*mazEF-mazF*stop or pRAB11-Pr-*mazEF*-*mazE*stop-*mazF*stop were cultivated as described above, with the exception, that 50 ml were harvested when reaching an OD_578_ of 2.5. In case of HG001Δ*spa* cells containing: pRAB11-*spa*, pRAB11-*spa*-mA, pRAB11-*spa-*mB, pRAB11-*spa*-mC or pRAB11-*spa-*mD, 25 ml were harvested after 2.25 hours. In order to determine cleavage of the *spa* transcript in different media, HG003 (pRAB11-*gfpmut2*), HG003Δ*mazEF* (pRAB11*-gfpmut2*) and HG003Δ*mazEF* (pRAB11-Pr-*mazEF*) were grown overnight (16 hours) in 10 ml chloramphenicol supplemented BHI, MHB and TSB each in 100 ml flasks. In the morning the cultures were inoculated into their respective media and either 16 ml (BHI and TSB) or 50 ml (MHB) harvested after 3.5 hours of growth. To determine cleavage of *rsbW*, cultures of HG003 (pRAB11-*gfpmut2*), HG003Δ*mazEF* (*pRAB11-gfpmut2*) and HG003Δ*mazEF* (pRAB11-Pr-*mazEF*) inoculated to an OD_578_ of 0.07 and then grown for another 3 hours were supplemented with KOH (30 mM final concentration) to increase transcript levels of the *rsbUVW-sigB* operon [31]. After 15 minutes of incubation, the cells were harvested.

Cultures of HG003 and HG003Δ*mazEF* carrying pRAB11-*rsbW*-trunc were inoculated in duplicates and of each strain, one sample was induced with ATc after 1.5 hours. After another hour of growth, cells were harvested. In combined experiments, applying both ATc and KOH, overnight cultures of HG003 and HG003Δ*mazEF* containing pRAB11-*rsbW*-trunc were first grown at 37°C in chloramphenicol supplemented medium. The next morning, three flasks of each strain were inoculated and one pair of HG003 and Δ*mazEF* was incubated at 42°C. After 3 hours ATc was added and the cells harvested after 1 hour. The rest of the cultures were incubated at 37°C. To one pair of those samples, ATc and KOH (30 mM) was added after 3 hours and the cells harvested 1 hour later (ATc and KOH simultaneously), to another 37°C pair of samples KOH was added after 1.5 hours and ATc after another 1.5 hours and the cells were harvested after 1 hour of incubation (KOH first, ATc later).

### RNA isolation and DNase I digests

RNA isolation and DNase I digests were done as described previously [36]. Briefly, the pellets were lysed in Trizol using a fast prep machine (MP bio FastPrep 24). The RNA was purified according to the manufacturer protocol and then taken up in 50 μl RNA storage solution. Per sample, 100 μg of total RNA was treated with 20 U of DNase I, the DNA free RNA was then purified by phenol/chloroform extraction following an ethanol precipitation and washing procedure. The RNA was finally taken up in 30 μl of DEPC treated ddH_2_O.

### Fluorescent *in vivo* primer extensions

Fluorescent primer extensions were performed as described by Schuster and Bertram [36] using 5–15 μg (35 μg in the case of *rsbVW*) DNA-free RNA. The gel images were adjusted with the curves tool in Photoshop (Adobe), then exported to 8 bit greyscale and labeled in Illustrator (Adobe).

### Determination of Protein A and RsbW expression by sfGFP fluorescence measurements

Cultures of HG003 and HG003Δ*mazEF* containing either the plamids pRAB11-Pr *spa*-*sfGFP* WT/mA/mD or pRAB11-*rsbV*-*rsbW*-*sfGFP* WT/mA/mC/mD were grown overnight in 10 ml BM, supplemented with chloramphenicol (10 μg/ml) at 37°C and 150 rpm. In the case of the *spa* constructs, GFP measurements were done immediately whereas the *rsbVW* constructs were first induced with 0.4 μM ATc for 2 hours and then measured. Cultures were inoculated to an OD_578_ of 0.07 from the overnight cultures in 50 ml BM with chloramphenicol in 250 ml flasks. In the case of the *spa* transcript, samples were measured after 3 and 5 hours after inoculation. The *rsbW* cultures were induced with ATc after one hour and GFP expression measured after 2 and 5 hours. 2 ml of each culture was harvested, the pellet washed in 1 ml PBS (pH 7.4) buffer and resuspended in 300 μl PBS. 100 μl samples were transferred in duplicates to a black, flat bottom 96 well plate and GFP levels measured at 525 nm (excitation at 485 nm) in a plate reader (Tecan) using the accompanying (Magellan) software. PBS buffer served as a reference to determine the background. All experiments from the same condition were done with the same amplification factor in biological triplicates and normalized to optical density.

### Autolysis assay

Cultures were grown in 10 ml TSB medium containing 10 μg/ml chloramphenicol. Cultures were then inoculated to an OD_578_ = 0.07 in 10 ml TSB without antibiotics. After two hours, an equivalent of OD_578_ = 1 was harvested and the pellet resuspended in 2 ml Tris/Triton buffer (50 mM Tris/HCl pH7.5, 0.05% Triton X-100). The samples were incubated shaking in cuvettes as 30°C and the OD_578_ measured every 30 minutes.

### MIC testing

For determination of MICs by Etest strips, *S*. *aureus* strains were grown overnight (16 hours) in 10 ml TSB, shaking (150 rpm) at 37°C in 100 ml baffled flasks and diluted the next morning to an OD_578_ of 0.1 in fresh TSB medium. 100 μl of diluted cells were plated on TSA plates and an Etest strip was placed on the agar with sterile forceps. Plates were incubated for 16 hours at 37°C. MIC tests in liquid culture were performed as described before [43].

### Growth experiments and antibiotic challenge in liquid culture

*S*. *aureus* strains were grown overnight in TSB medium as described above. For growth curves of untreated cells, cultures were inoculated to an OD_578_ of 0.07 in in 100 ml baffle flasks from the overnight cultures and incubated shaking at 150 rpm. At time points indicated, 100 μl samples were withdrawn, washed with 100 μl sterile saline solution (0.85%) and 10μl of serial dilutions per sample were dropped onto TSA plates and incubated overnight at 37°C to determine the number of colony forming units per ml of culture.

For the penicillin G or oxacillin challenge, the cells were precultured as above in flasks, but after 3.5 hours transferred to 14 ml round bottom conical tubes in TSB medium with a final volume of 2.14 ml and incubated shaking at 150 rpm. The addition of penicillin G or oxacillin was defined as time point zero. After sample withdrawal at time point zero, 100 μl of penicillin G or oxacillin in appropriate stock solutions was added to the cultures and samples taken in defined intervals. At each time point, 100 μl samples were withdrawn, washed, diluted and dropped onto TSA plates as above. Percentage of survival was calculated from cfu/ml. Shown are the averages and standard deviations of at least three biological replicates per experiment. The p value was calculated by a paired two-tailed student’s t-test.

## Results

### *spa* transcript produces band in primer extension experiments at UACAU *in vivo*

**Fig 1:**
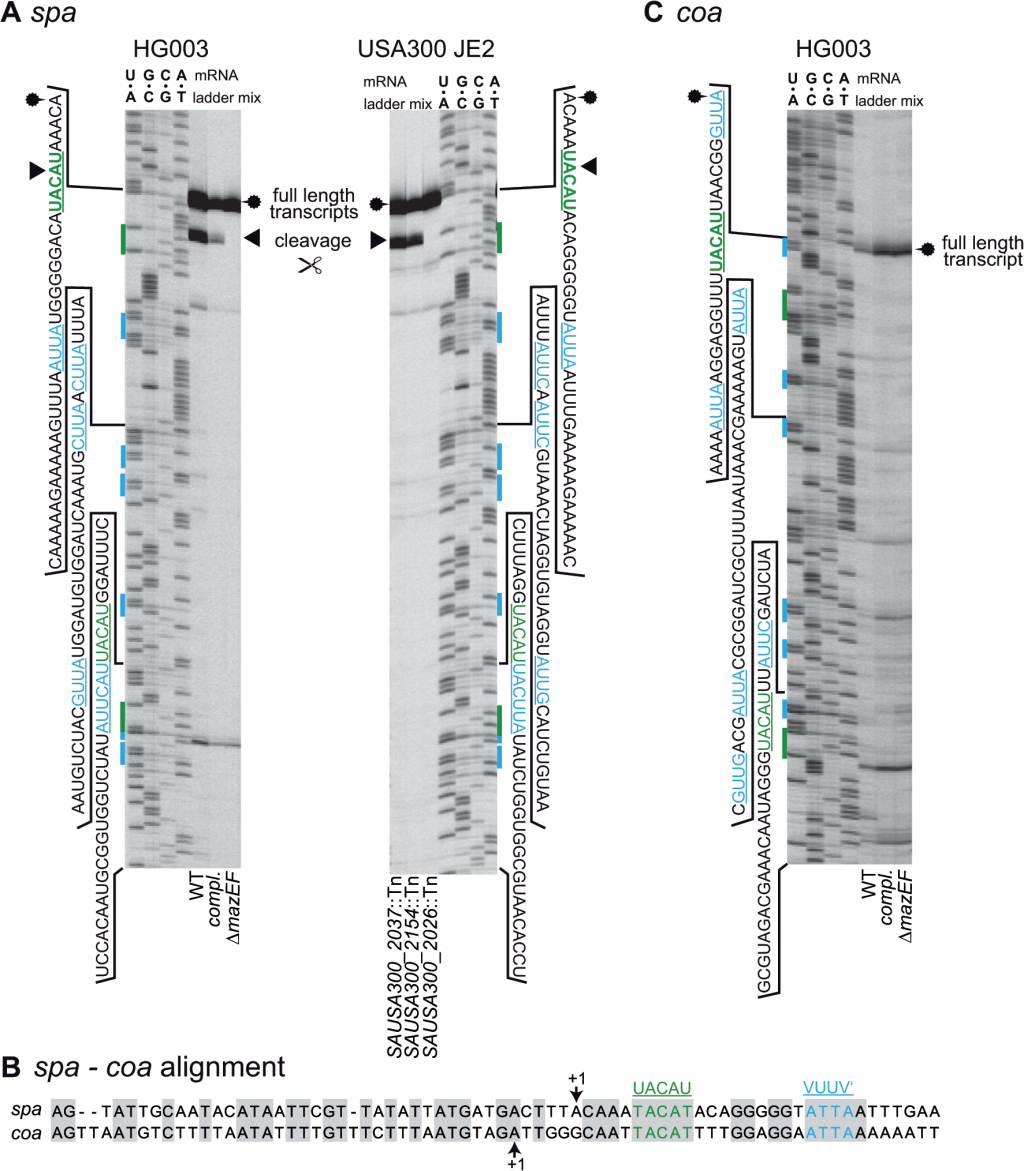
Cleavage of transcripts isolated from *S*. *aureus*. **(A) Cleavage of *spa* transcript in *S*. *aureus* HG003 and NARSA transposon mutant library derivatives of strain USA300 JE2**. The *spa* transcript was cleaved *in vivo* at the first 5’ UACAU site (green) in HG003 and the complemented *mazEF* mutant. Cleavage did not occur at the VUUV’ sites (blue) and in the *mazEF* deletion mutant. Cleavage was also visible at the first UACAU site but not at VUUV’ in the transposon mutant strains NE565 (RNA helicase, SAUSA300_2037) and NE1535 (galactose-6-phosphate isomerase, SAUSA300_2154). The UACAU site is not cut in the *mazF* mutant strain NE1833 (SAUSA300_2026). RNA sequences of sequencing ladders are shown on the sides of the gels. Putative cleavage sites are marked in the sequence and sequencing ladders by green and blue text and bars. Cleavage is denoted by a black arrow head and the full length transcript is indicated by a fuzzy circle. **(B) Alignment of the transcript and upstream region of *spa* and *coa*.** The sequences of *spa* and *coa* possess conserved UACAU and VUUV’ sites. Identical bases are shaded in gray. +1 denotes TSP. **(C) Test for MazF-dependent cleavage of *coa* transcript.** Cleavage at UACAU or VUUV’ could not be observed in any of the examined strains despite high similarity to the *spa* transcript. WT = HG003 (pRAB11-*gfpmut2*), Δ*mazEF* = HG003Δ*mazEF* (pRAB11-*gfpmut2*), compl. = HG003Δ*mazEF* (pRAB11-*P*_*mazEF-*_ *mazEF*).

A markerless *S*. *aureus* HG003 deletion mutant of the *mazEF* locus (SAOUHSC_02303- SAOUHSC_02304) was generated while keeping the P_*mazEF*_ promoter and a weak transcriptional terminator downstream of the *mazEF* locus intact. We first investigated, whether the transcript of the *spa* gene (encoding Staphylococcal Protein A) was cleaved in this mutant, as *spa* mRNA was previously suggested as a substrate for MazF [26]. RNA prepared from exponential growth phase *S*. *aureus* HG003 and the *mazEF* deletion strain with or without a complementation plasmid was used for primer extension experiments probing the *spa* transcript (Fig 1A, left panel). The transcriptional starting point (TSP) of the *spa* gene, 12 bp downstream of the -10 region of the promoter was confirmed as published previously [44]. A second band was observed at the most upstream UACAU site close to the 5’ end of the transcript only in HG003 and the complemented Δ*mazEF* strain. No signals indicating cleavage were visible at a second UACAU site, as well as at six VUUV’ sites present in the analyzed region. Further bands observed about 20 and 100 bps downstream of the TSP in all three strains were interpreted as non-specific reverse transcription termination products. A similar banding pattern was observed in the plasmid-free strains HG003 and Δ*mazEF* in medium without antibiotics (see S1 Fig). Interestingly, the banding pattern at UACAU was observable in wild-type (WT) HG003 also in the presence of an intact *mazE* gene, whereas cleavage in previous publications had relied on overexpression of MazF [26].

To confirm the potential cleavage of the *spa* transcript at the 5’ located UACAU site, we repeated the experiment with three mutants from the NARSA library [45] that carry transposons either in *mazF* (SAUSA300_2026) or two different genes (SAUSA300_2013 and SAUSA300_2154), which both do not encode TA systems. The mutant affected in *mazF* did not produce a primer extension band at UACAU, whereas the other two strains showed an identical cleavage pattern to HG003 and the complemented mutant (Fig 1A, right panel).

According to literature and a ClustalO alignment, the 5’ ends and promoter regions of the *spa* and the *coa* (coagulase) transcripts of *S*. *aureus* are similar, and both contain a UACAU and a VUUV’ site shortly downstream of the +1 position (Fig 1B). Also, the expression of both proteins is repressed by the regulatory RNAIII molecule binding to the transcripts [44,46].

Therefore, we investigated if primer extension also yields comparable signals with the *coa* transcript at the UACAU site (Fig 1C). Except the full-length transcript band that was visible in the primer extension experiment in all strains, we observed no additional signals that would indicate specific cleavage of *coa* in the WT and the complemented mutant.

### The *spa* transcript is cleaved in a *mazEF*-dependent fashion

The primer extension band we observed at the UACAU site of the *spa* transcript had previously been ascribed to an alternative TSP [47]. We hence investigated the possibility that an as yet unidentified additional downstream promoter may also drive *spa* transcription. If the band at UACAU was caused by an additional promoter, the exchange of the region upstream of *spa* should abolish this primer extension band.

**Fig 2:**
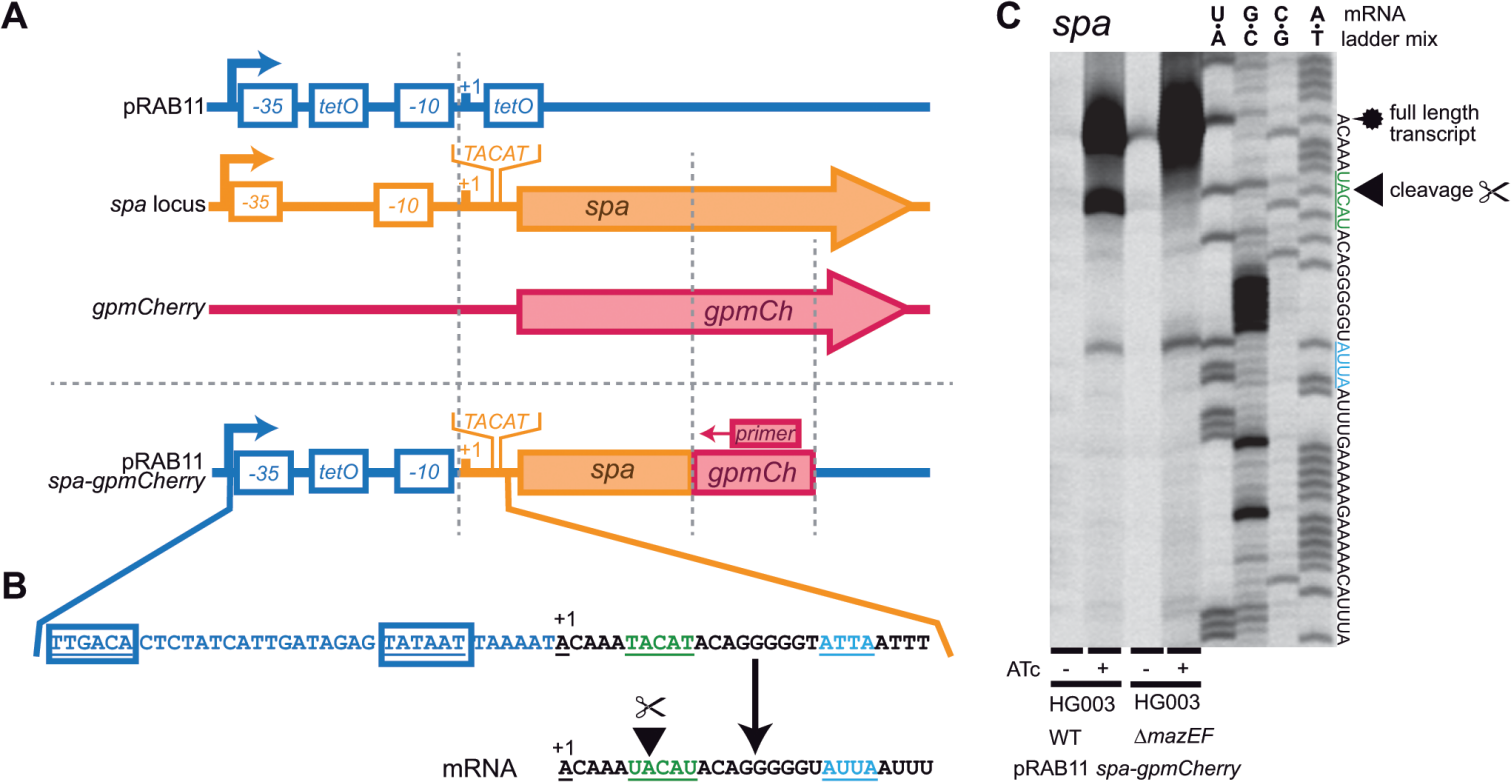
Replacement of native *spa* promoter with P_*xyl/tet*_ to distinguish cleavage from alternative transcriptional starting points. **(A) Plasmid construction of pRAB11-*spa-gpmCherry***. The first three elements depict source DNA fragments and the last element the final vector. The +1 position of the 5’ end of the *spa* transcript was cloned into position +1 of the ATc inducible pRAB11 plasmid. When induced, the resulting transcript is expected to be identical to that from the native *spa* promoter. Part of *gpmCherry* was integrated into the sequence to enable specific primer binding to the chimeric transcript and therefore eliminate the native *spa* transcript background. Promoters are depicted as angled arrows, promoter elements as white filled boxes, genes as arrows. **(B) Close-up DNA sequence of promoter and transcript region of pRAB11-*spa-gpmCherry*.** Promoter region (-35 and -10) shown in blue, *spa* transcript in black and green. The first UACAU is underlined and marked in green and the first VUUV’ site is marked in blue. **(C) Primer extension result of HG003 and Δ*mazEF* containing pRAB11-*spa-gpmCherry*.** Full length transcripts (fuzzy circle) were strongly visible upon induction with ATc. The signal at UACAU (arrowhead) occurred only in the HG003 WT, but not in the *mazEF* deletion mutant strain indicating actual cleavage.

First, we determined the TSP of the P_*xyl/tet*_ promoter of plasmid pRAB11 [48], which is regulated by the repressor TetR and inducible by ATc (S2 Fig). Then, we constructed a pRAB11 derived plasmid which contained the 5’ end of the *spa* transcript in such a way that the *spa* promoter was removed and the +1 positions of the P_*xyl/tet*_ promoter and the native *spa* transcript were congruent (Fig 2A). In addition, a short sequence of a Gram positive (*gp*) adapted *mCherry* gene was transcriptionally fused to *spa*, solely to provide a specific primer binding site. The resulting plasmid (Fig 2B) was then used to transform HG003 and the Δ*mazEF* strain. By using these constructs, we were able to ignore the endogenous *spa* transcript and to reproduce the 5’ end of the *spa* mRNA.

In the absence of the inducer ATc, only weak bands of the full length *spa-gpmCherry* hybrid transcript were visible, whereas the intensity increased drastically when ATc was added (Fig 2C). Specific cleavage of the UACAU site within the *spa-gpmCherry* transcript was clearly visible in HG003 but absent in the *mazEF* deletion strain. Other minor bands were judged as unspecific, since they occurred in both the WT and the Δ*mazEF* mutant. These results confirmed that the 5’ terminus located at the UACAU site of *spa* is caused by cleavage and not by an additional promoter.

### MazF is active even in the presence of MazE under non-stress conditions

Under standard conditions most toxins from chromosomal TA systems are assumed to be inhibited by the cognate antitoxin, only to become active under stress. However, MazF cleavage at the UACAU site of *spa* mRNA in our experiment occurred despite the presence of an intact *mazE* gene. In previous *in vitro* tests, purified MazF exhibited endoribonuclease activity [24,26,29]. We next aimed to demonstrate that MazF alone was sufficient for *in vivo* cleavage in *S*. *aureus*.

**Fig 3:**
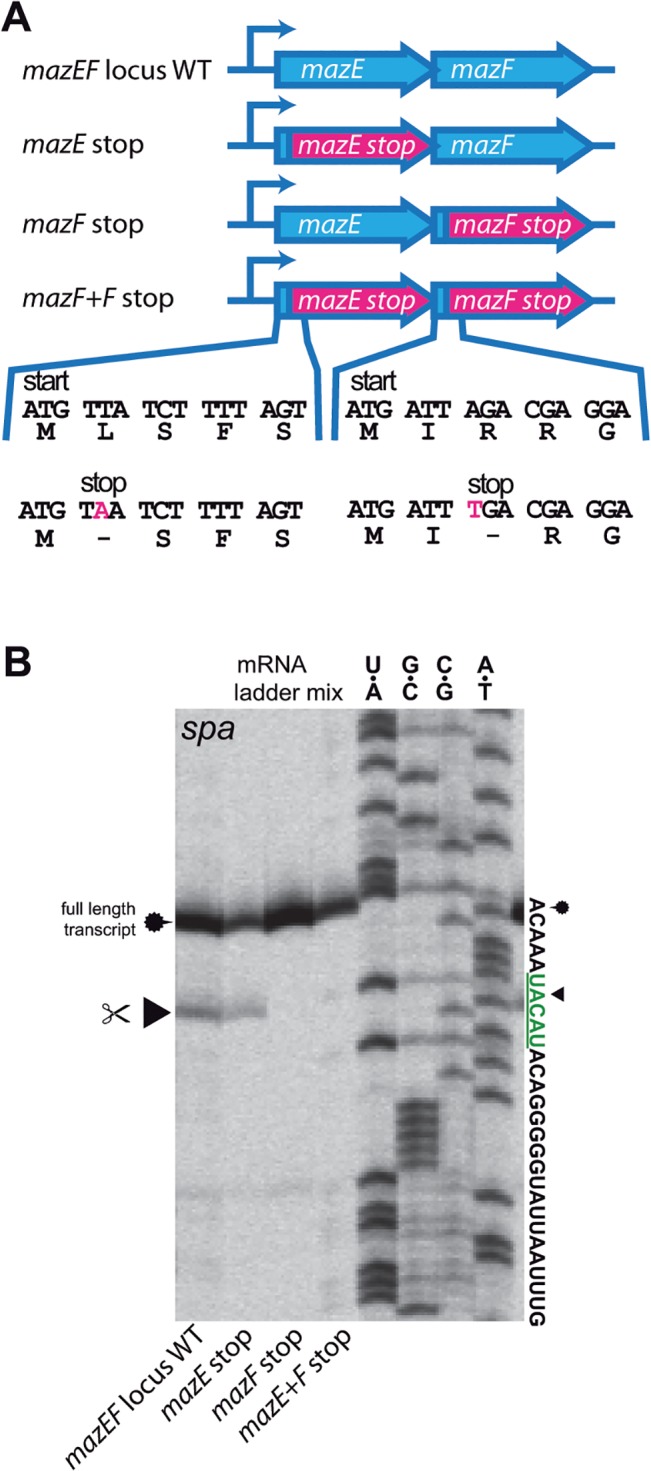
Identification of *mazEF* components critical for *spa* cleavage. **(A) Overview of pRAB11 constructs containing the native P**_***mazEF***_ **promoter and full-length or truncated *mazEF* genes.** Stop codons were introduced shortly after the start codons of either *mazE*, *mazF* or both. Nucleotide and amino acid sequences of WT and mutants are depicted and mutations marked in pink. **(B) Primer extension experiment of pRAB11-*mazEF* variants in HG003 Δ*mazEF*.** Cleavage at UACAU in the *spa* transcript occurred when *mazF* was intact, even in the presence of *mazE*.

Therefore, we modified the complementation plasmid used in previous experiments, containing the native promoter and the full length *mazEF* locus and introduced premature stop codons in either *mazE*, *mazF* or both genes (Fig 3A). When we investigated HG003Δ*mazEF* transformed with the resulting plasmids, cleavage at the UACAU sequence of the *spa* transcript was observed with intact *mazF*, irrespective of the absence or presence of *mazE* (Fig 3B).

To explore the possibility that the observed cleavage only occurred under the specific conditions used in our setup, we repeated the primer extension assay with cells cultured in different staphylococcal growth media, leaving standard laboratory conditions unaffected (37°C, 150 rpm).

**Fig 4:**
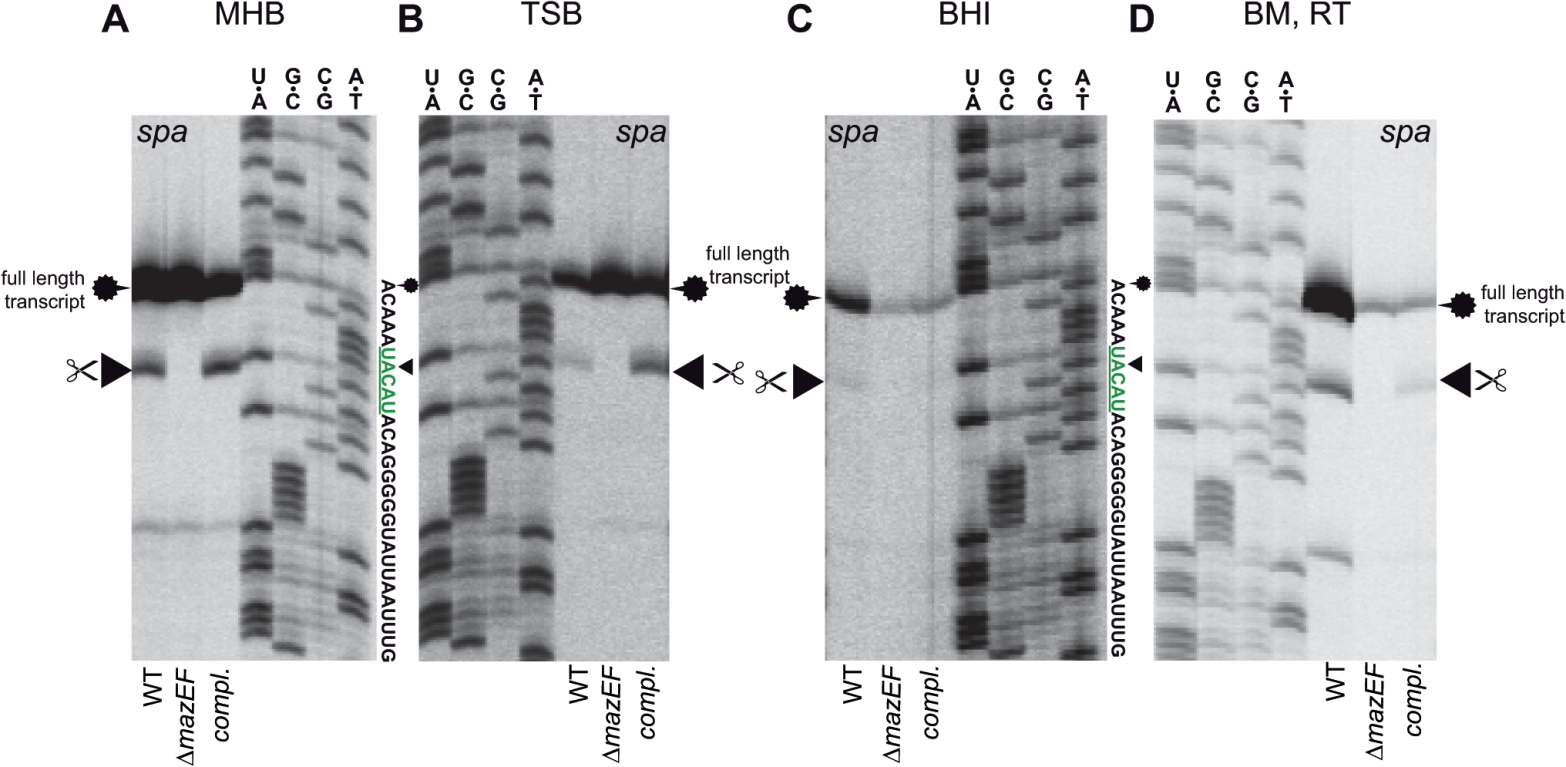
Cleavage capability of *S*. *aureus* MazF in the presence of *mazE*, dependent on growth in different media. **(A) MHB: Mueller-Hinton-Broth**, **(B) TSB: Tryptic soy broth, (C) BHI: brain heart infusion medium**, **(D) BM: basic medium and sample preparation without any cold-shock inducing steps**. The *spa* transcript is cleaved at UACAU (arrowhead) by MazF in the presence of *mazE* in HG003 and in the complemented Δ*mazEF* mutant in all tested media. Cleavage was absent in the Δ*mazEF* mutant. Cleavage band intensity at UACAU in cells grown in BHI medium was weaker than from cells grown in other media but correlated to reduced *spa* transcript levels in BHI medium in these experiments. WT = HG003 (pRAB11-*gfpmut2*), Δ*mazEF* = HG003Δ*mazEF* (pRAB11-*gfpmut2*), *compl*. = HG003Δ*mazEF* (pRAB11-*P*_*mazEF*_ *-mazEF*).

Cells cultured in MHB produced strong full-length *spa* transcript bands. In addition, a clear cleavage of the UACAU site was visible in HG003 and the complemented Δ*mazEF* mutant (Fig 4A). The strains grown in TSB also produced high amounts of full length transcripts and cleavage at the UACAU site in the WT and complemented mutant was evident (Fig 4B). When the cells were grown in BHI, the amount of full length *spa* transcript was greatly reduced in comparison to BM or TSB medium (Fig 4C). The band indicating cleavage at UACAU was also less pronounced but still visible in the WT strain.

During the primer extension sample collection and preparation procedure, the cells are subjected to a number of harsh thermal shifts. Although potential cold-shock induced transcriptional repression would not explain MazF activation, we wanted to rule out that the temperature differences during cell harvest had influenced MazF activity. We therefore repeated the previous experiment in BM medium without any incubation steps on ice or freezing of the cells. These gently treated cells showed exactly the same UACAU cleavage pattern as in the previous experiments although total transcript amounts were slightly reduced in the *mazEF* mutant strain (Fig 4D).

### *S*. *aureus* MazF cuts the UACAU sequence with high specificity

To date, the exact *in vivo* cleavage specificity of *S*. *aureus* MazF has not unambiguously been shown, as most experiments had been performed *in vitro* or in *E*. *coli* [24,26,29]. We were curious if MazF also cuts mutated UACAU sites *in vivo* in *S*. *aureus*.

**Fig 5:**
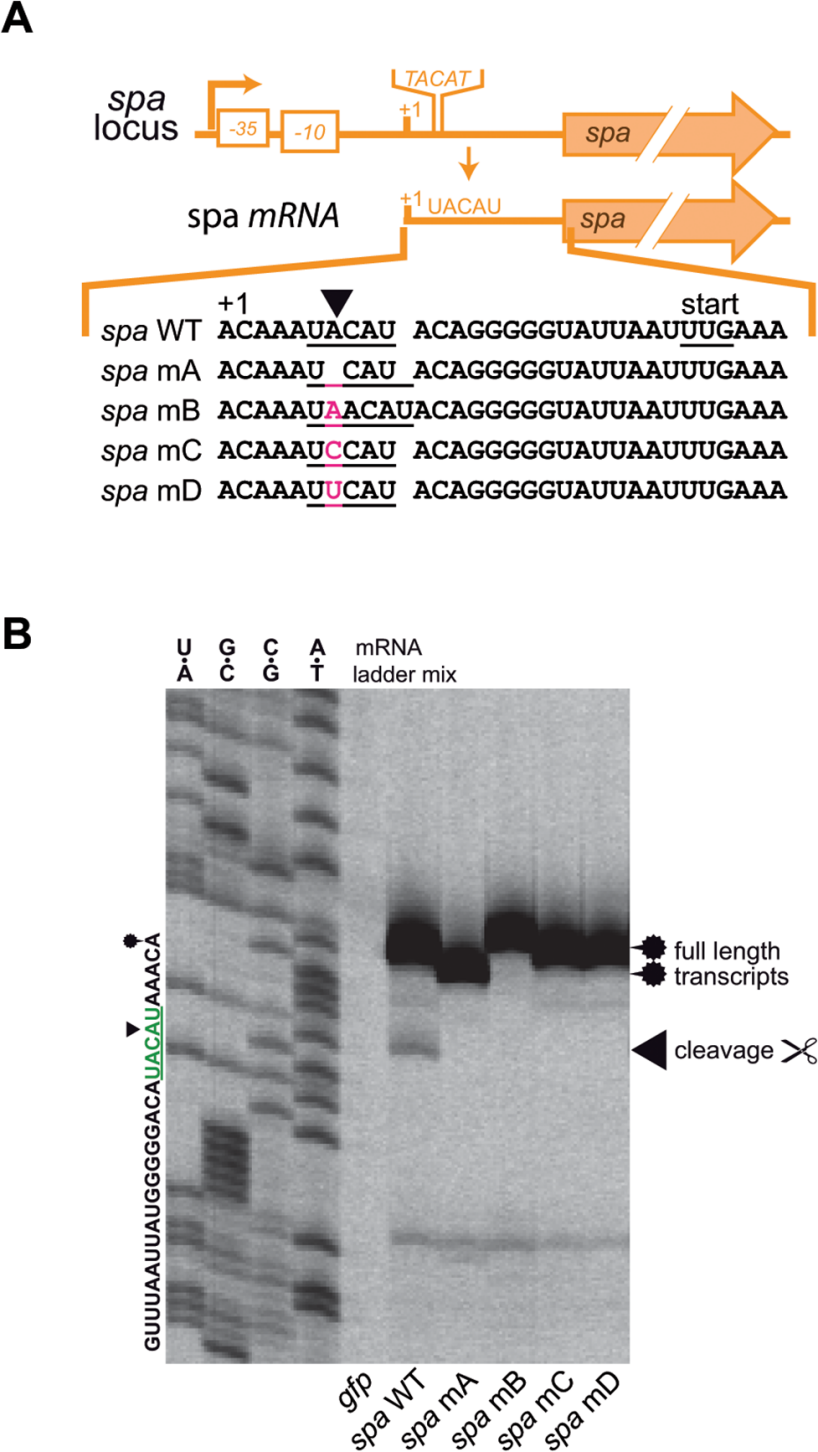
Sequence aberrations of the UACAU site in the *spa* transcript to elucidate MazF sequence specificity. **(A) Native *spa* promoter, transcript and UACAU sequence mutations.** UACAU site is underlined in transcript sequence and mutated bases are marked in pink (mA: insertion, mB: deletion, mC: transversion, mD: transversion to create a VUUV’ sequence). **(B) Primer extension results of *spa* UACAU derivatives subjected to MazF treatment *in vivo*.** Cleavage (arrowhead) of *spa* was only observed in the UACAU sequence, whereas any changes (including that to VUUV’) abolished cleavage. The shift of the full length transcript band (fuzzy circle) in the mA and mB strains is due to the insertion and deletion of one base each. All plasmids were contained in HG001Δ*spa* to eliminate background from WT *spa* transcript. *gfp* stands for pRAB11-*gfpmut2*; *spa* WT for the native *spa* transcript and mA-D for the respective pRAB11 constructs from **(A)**.

To address this question, plasmids containing the complete *spa* locus including the native promoter were constructed. Single base deletions, insertions or substitutions at the first UACAU site of the *spa* transcript were introduced (Fig 5A) to modify the putative recognition site and in one case generating an AUUC (= VUUV’) motif. The resulting plasmids and a *gfp* control were used to transform *S*. *aureus* HG001Δ*spa* cells to remove any background from endogenous *spa* transcripts. *S*. *aureus* HG001 is largely isogenic to HG003 [35] differing in a repaired *tcaR* regulator.

As expected, HG001Δ*spa* carrying the control plasmid, produced no signal in the primer extension gel (Fig 5B). Strains harboring the WT and mutated *spa* plasmids produced strong bands of full length mRNA transcripts. Cleavage was only observable in the WT *spa* construct, which underscores a strict target specificity of MazF for the UACAU sequence.

### The *rsbW* transcript is also cut by MazF *in vivo*

Despite the cleavage of *spa* mRNA, not all transcripts containing UACAU sites (such as *coa*, see above) appear to be substrates of *S*. *aureus* MazF. We aimed to find out whether other mRNAs might as well be cleaved by MazF in *S*. *aureus*. In a previous report [26], a decrease in *sigB* transcript levels upon *mazF* overexpression had been determined, but whether this mRNA was directly cleaved or downregulated in another way was not investigated. Due to its genomic association with *mazEF* throughout the staphylococcal genus (S3 Fig), we studied the *rsbUVW-sigB* region for possible MazF-dependent cleavage.

**Fig 6:**
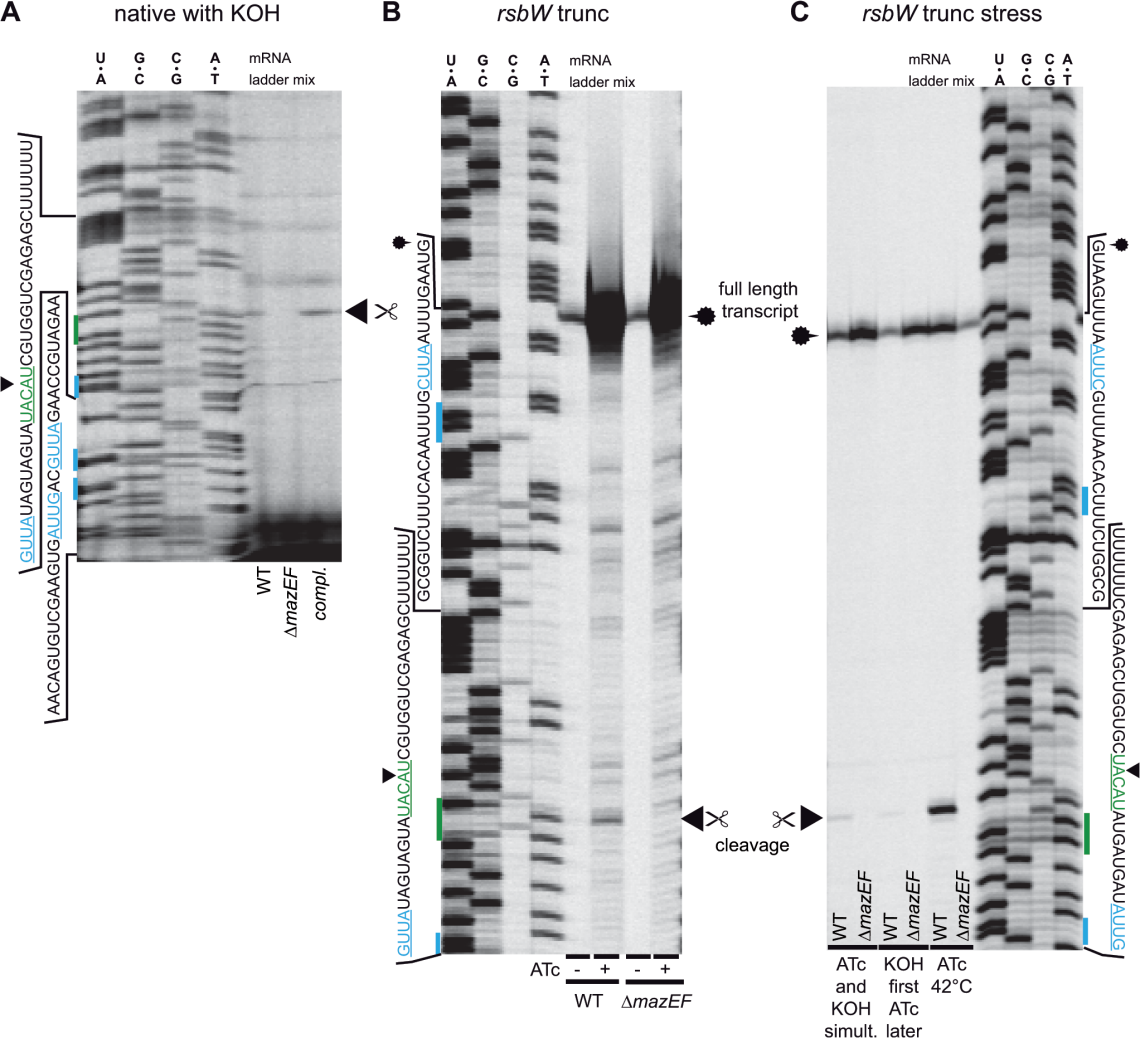
Cleavage of *rsbW* transcript *in vivo*. **(A) native *rsb* transcript.** To increase transcript levels, 30 mM KOH was added to the culture. Slight cleavage bands could be seen at UACAU only in HG003 and the complemented Δ*mazEF* mutant. **(B) Artificially truncated *rsbW* transcript.** 5’ ends of transcripts became visible in both strains upon ATc induction (loading control) due to truncation. Bands indicating cleavage at UACAU were visible in HG003 but not in the Δ*mazEF* mutant. **(C) Artificially truncated *rsbW* transcript with KOH stress and heat shock.** Growth at 42°C increased levels of cleaved *rsbW* transcript at UACAU.

Cells of HG003, Δ*mazEF* and the complementation mutant were grown to exponential phase, *sigB* expression was stimulated by the addition of KOH (30 mM) and primer extensions were performed using the isolated total RNA with different primers probing the UACAU sites of the *rsbUVW*-*sigB* locus. Cleavage bands at the UACAU site of the *rsbW* transcript could be observed in HG003 and the complemented strain, but not in the *mazEF* mutant (Fig 6A). Cleavage of other UACAU sites in the *rsbUVW-sigB* locus was not identified. Due to the low amounts of cleavage products in the total RNA sample, we constructed an ATc-controllable overexpression plasmid containing a truncated *rsbW* transcript, which also served as a loading control producing a band in the vicinity of the UACAU site (Fig 6B). Upon induction, large amounts of transcripts were present in both strains and cleavage at UACAU occurred in HG003 but not in Δ*mazEF*. In search for factors that stimulate the RNase activity of MazF, we used heat-shock and KOH stress to influence σ^B^ activity. Indeed, cleavage activity of MazF was increased at 42°C (Fig 6C).

### Transcript cleavage does not change expression of Protein A or RsbW

We next investigated, whether cleavage of the UACAU sites in the transcripts of *spa* and *rsbW* might influence expression of the encoded products Protein A or RsbW, respectively. In the case of *spa*, the UACAU site is located several bases upstream of the start codon and although removal of the RNA 5’ end would not interrupt the coding region, regulation of *spa* by RNAIII and RNase III might be affected [44]. On the other hand, the UACAU site of the *rsbW* transcript is located within the open reading frame, suggesting a decrease in RsbW amounts when cut. Translational fusions of Protein A or RsbW with a superfolder (sf) GFP variant [49,50] and modified UACAU sites were created as a readout to monitor the putative post-transcriptional control exerted by MazF on protein levels.

**Fig 7:**
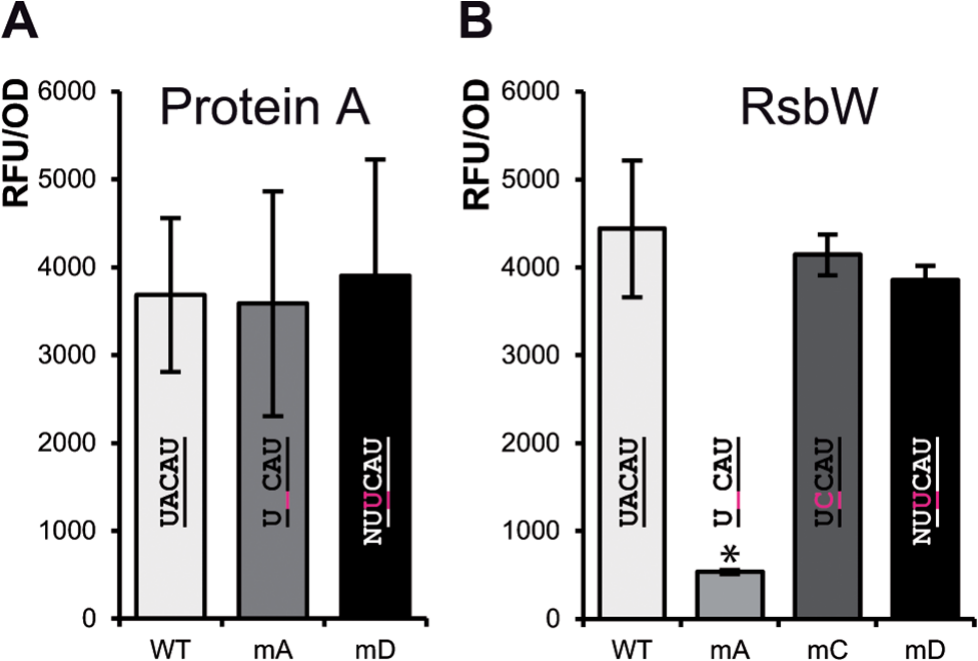
Cleavage of *spa* and *rsbW* transcript by MazF does not significantly influence levels of Protein A and RsbW. Fluorescence measurements of HG003 with plasmids producing transcripts of *spa* and *rsbW* transcripts translationally fused to superfolder green fluorescent protein (*sfgfp*) gene. Results obtained with respective Δ*mazEF* strains can be found in S4 and S5 Figs. **(A) Fluorescence of HG003 cells carrying a *spa*-*sfgfp* fusion construct.** No significant differences were detectable in fluorescence between WT and the UACAU mutants after 5 hours of growth. **(B) Fluorescence of HG003 cells carrying a *rsbW*-*sfgfp* fusion construct.** Fluorescence of the mA strains was greatly reduced, due to the one base deletion leading to a frame-shift. The UACAU mutants mC and mD did not lead to a significant decrease in fluorescence. Shown are averages and standard deviations from at least three independent experiments and the respective UACAU variant sequences. Asterisk (*) denotes a significant difference to the HG003 WT variant fluorescence based on a student’s t-test (p<0.05).

When grown for 5 hours, the change of the UACAU site to UCAU (mA) or UUCAU, generating a VUUV’ motif (mD), did not significantly change the fluorescence, indicating unchanged Protein A levels in HG003 (Fig 7A). Results were similar when fluorescence was measured directly from the overnight culture (A in S4 Fig), after three hours of incubation (B in S4 Fig) or by incubating the overnight culture at 42°C instead of 37°C (D and E in S4 Fig). In none of the cases tested did changes of the UACAU site increase fluorescence in the HG003 background. In fact, at 42°C, fluorescence decreased significantly when the UACAU site was damaged (D and E in S4 Fig)

Similar results were observed, when the RsbW-sfGFP samples were analyzed. Fluorescence intensities of HG003 were similar also when the UACAU site was changed to UCCAU (mC) or UUCAU (mD) (Fig 7B). The low fluorescence in the UCAU (mA) construct is due to the frame shift caused by the one base deletion and thus represents the basal level. When fluorescence of cells was measured from overnight cultures or cultures grown for 3 or 6 hours, at 37°C or 42°C, respectively, results were in general similar (A-F in S5 Fig). No significant changes were detectable compared to the WT construct in HG003 when the UACAU site was changed, with the exception of the shorter mA variant.

In summary, change of the UACAU sites to a non-cleavable sequence in the *spa* and *rsbW* transcripts did not seem to increase abundances of Protein A or RsbW. Judging from these experiments, MazF does not discernibly regulate these loci by mRNA cleavage.

### *S*. *aureus* Δ*mazEF* is more sensitive to β-lactam antibiotics

In search for additional phenotypes, we observed the previously published increase in staphyloxanthin production and autolysis behavior [25] of our *mazEF* deletion mutant, but these phenotypes could not be complemented (S6 Fig). In terms of growth behavior, the cfu/ml count between the WT and the *mazEF* deletion mutant was not significantly different, as assayed on an hourly basis up to 24 hours (S7 Fig).

**Fig 8:**
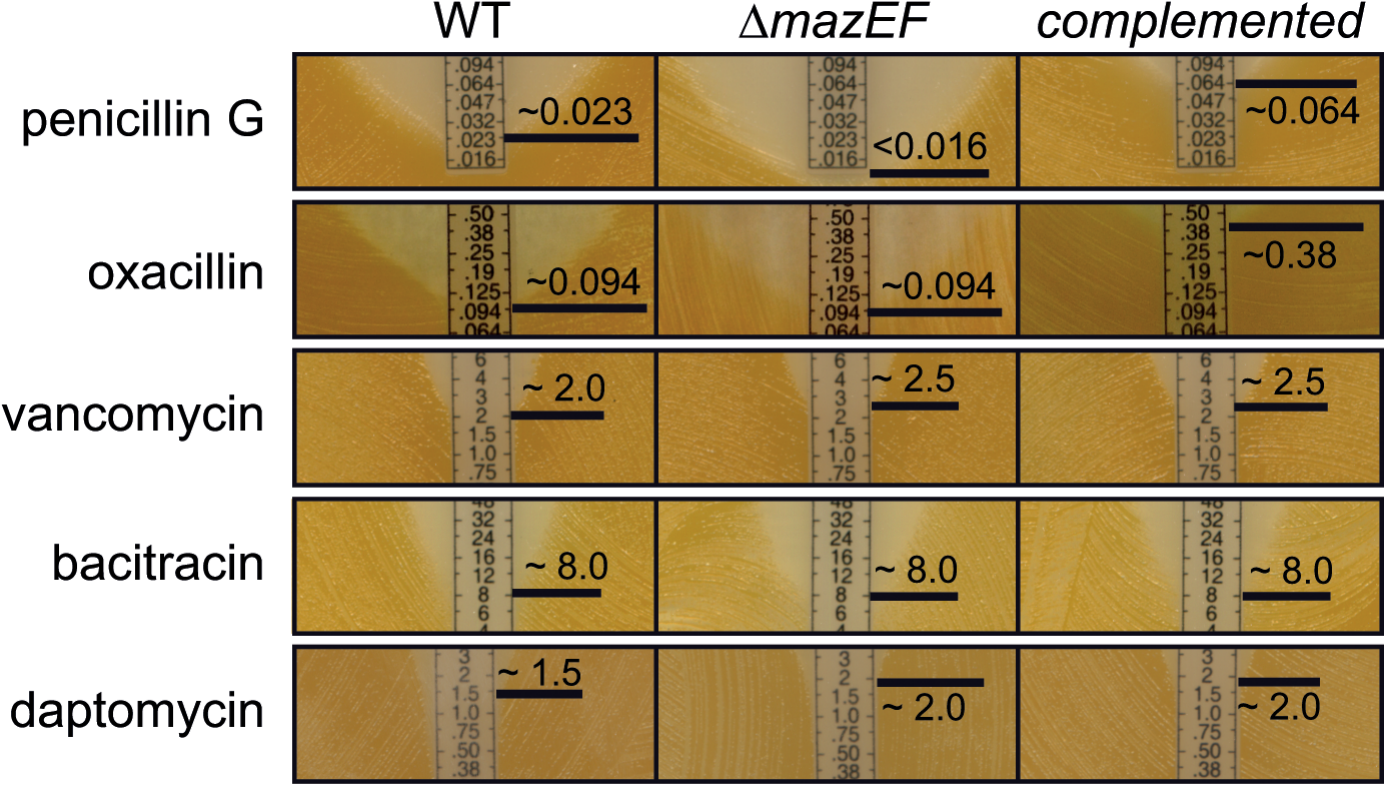
Sensitivity of HG003, Δ*mazEF* and complemented mutant to different antibiotics. MIC was determined by plating cells on solid media and placing Etest strips on bacterial lawn. The experiments for each antibiotic were done at least in duplicates. The MIC of penicillin for Δ*mazEF* was lower than for the WT and much higher in the complemented mutant. In the case of oxacillin, an increase in the MIC was observable for the complementation mutant in comparison to the WT and the Δ*mazEF* strain. In contrast, the MICs of vancomycin, bacitracin and daptomycin were almost identical between strains. WT = HG003 (pRAB11-*gfpmut2*), Δ*mazEF* = HG003Δ*mazEF* (pRAB11-*gfpmut2*), *complemented* = HG003Δ*mazEF* (pRAB11-*P*_*mazEF*_ *-mazEF*).

As the *mazEF* TA system is upregulated by antibiotic stresses, we decided to determine the susceptibility of HG003, Δ*mazEF* and the complemented strain to a number of drugs. As judging from Etest strips, the MIC of penicillin for HG003 (pRAB11-*gfpmut2*, used as a control) was about 0.023 μg/ml, whereas the deletion mutant with the same control plasmid exhibited an MIC of less than 0.016 μg/ml (Fig 8, top row). Plasmid-dependent complementation of the *mazEF* operon over-compensated the effect, by increasing penicillin MIC to 0.064 μg/ml. A similar kind of over-compensation was observed in the complementation mutant with oxacillin (Fig 8, 2^nd^ row).The MICs of other tested antibiotics, namely vancomycin, bacitracin and daptomycin were barely different between strains (Fig 8, starting from 3^rd^ row).

**Fig 9:**
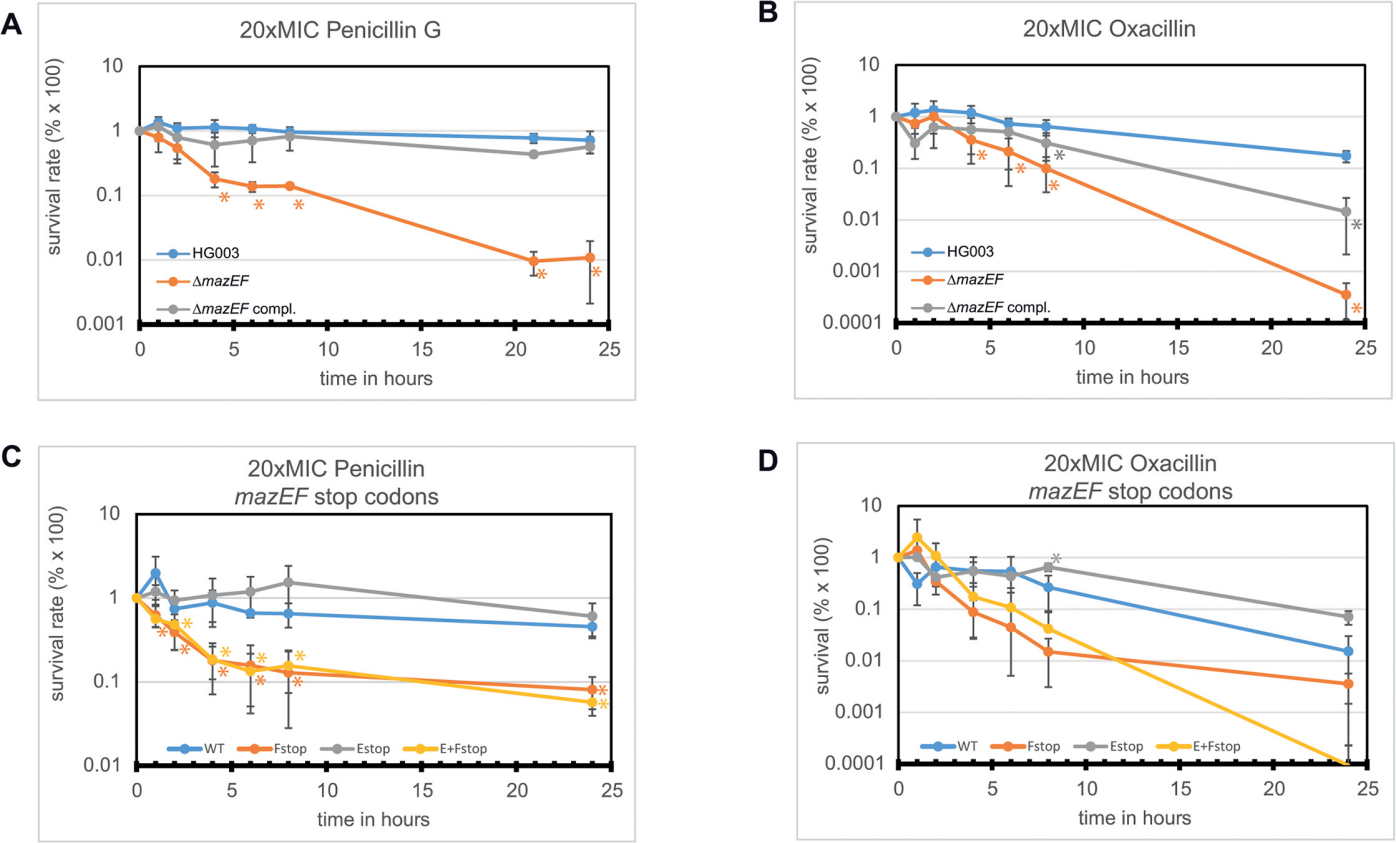
Time dependent eradication of cultures when challenged with 20× the MIC of penicillin or oxacillin in exponential phase. **(A)+(B). Survival of WT (HG003), Δ*mazEF* containing pRAB11 gfp and the complemented mutant in the presence of penicillin (A) or oxacillin (B).** Δ*mazEF* was significantly more sensitive (about 2 logs) to penicillin compared to WT (orange asterisks). The complemented mutant was not significantly different from the WT. When challenged with oxacillin the differences were not as significant, but the overall picture was the same as for penicillin. Asterisk (*) denotes a significant difference to the HG003 WT variant fluorescence based on a student’s t-test (p<0.05). **(C)+(D). Survival of Δ*mazEF* when complemented with different constructs in the presence of penicillin (C) or oxacillin (D).** The pRAB11-P_*mazEF*_-*mazEF* derivatives from Fig 3A were used in this experiment. When *mazF* alone or together with *mazE* was disrupted, penicillin sensitivity was significantly (yellow and orange asterisks) higher than the WT. Disruption of *mazE* only did not lead to a significant change in penicillin sensitivity. In the case of oxacillin the differences were not as obvious but in general similar to penicillin treatment. Shown are averages and standard deviations from at least three independent experiments. 20× MIC refers to the MIC of the WT.

The penicillin susceptibility phenotype was also investigated in liquid media using exponential growth phase (3.5 hours) cultures of HG003, Δ*mazEF* and the complemented mutant. The cultures were challenged with 20-fold the MIC of penicillin of the WT and the CFUs were assayed over a 24 hour period (Fig 9A). The survival of the complemented *mazEF* mutant and the WT was not significantly changed. In contrast, the *mazEF* deletion mutant was severely more susceptible and its viable cell count was significantly reduced by almost 100-fold compared to the WT or the complemented mutant after 24 hours, in agreement with the previous MIC experiments. When we tested the clinically relevant β-lactam antibiotic oxacillin (Fig 9B), a similar pattern as for penicillin treatment was observable, although all cells including the WT were slightly more susceptible to the antibiotic.

To determine if the penicillin sensitivity phenotype was linked to *mazE*, *mazF*, or both genes, we used the stop codon constructs already employed in a previous experiment (Fig 4) to repeat the 20×MIC penicillin experiment (Fig 9C). When both *mazE* and *mazF* genes used for complementation were intact, the survival only slightly declined over a 24 hour period. A similar curve progression as the WT was also observable with the full-length *mazF* gene and truncated *mazE*, but overall slightly higher. When the *mazF* gene alone or simultaneously with *mazE* was inactivated, the survival was strongly impaired, indicating that *mazF* is critical for penicillin susceptibility. Tests with oxacillin yielded a similar, albeit not significant outcome (Fig 9D).

## Discussion

In this work, we provide new insights into mechanisms and influences of the *S*. *aureus mazEF* TA system *in vivo*. Previously, two research groups had proposed different RNA cleavage specificities for MazF [24,26,29] but except for one experiment in *S*. *aureus*, most of the studies had been conducted either *in vitro* or in *E*. *coli*. Ectopic overexpression of *mazF* had resulted in decreased amounts of *spa*, *sigB* and additional transcripts, strongly indicating cleavage by MazF [26]. Targeting of the *ctpA* transcript at VUUV’ in *S*. *aureus* had also been outlined, albeit with weak cleavage activity [24].

In this study, we could unambiguously demonstrate that the *spa* and *rsbW* transcripts are cleaved *in vivo* in *S*. *aureus* by the TA system component RNase MazF. Results from previous work done in *E*. *coli* [29] are in line with our data showing that UACAU is the main cleavage site of the *S*. *aureus* MazF *in vivo*. Change of the UACAU site to VUUV’ of the *spa* transcript, as well as single mutations or deletions, abolished MazF dependent cleavage in *S*. *aureus*. It is conceivable that such mutations render the RNA target site structurally unsuitable for MazF cleavage. However, due to the fact that another UACAU site located within the *rsbW* transcript is also cut, we assume that cleavage is specific to the sequence. In the case of *spa*, promoter exchange experiments demonstrated that the primer extension band at UACAU is in fact a result of cleavage and not caused by a secondary promoter as previously proposed [47].

According to the results from translational reporter fusions, MazF-dependent mRNA-cleavage did not influence the abundances of the encoded Protein A or RsbW, which may indicate a role of MazF in fine-tuning of gene expression that was below the threshold of quantification using our setup. Nevertheless, the *spa* transcript can be used as an *in vivo* readout for MazF cleavage, aside from known *in vitro* reporter systems [51]. In addition, *spa* transcript levels are exceptionally high compared to other transcripts and thus are easy to detect in primer extensions.

It remains unclear why cleavage does not occur at all UACAU sites of RNAs. As proposed previously, specific transcripts may be protected by RNA binding proteins that mask access to the cleavage sites [26]. Presumably, the staphylococcal transcriptome adapted to the presence of MazF and transcripts that should not be controlled by this RNase are either protected [26], evolved to eliminate cleavage sites [29] or cleavage is compensated by higher transcript levels.

Elucidating the entirety of MazF cleaved RNAs is the next step in characterizing the *mazEF* TA system. Advances in next-generation RNAseq approaches [52] can aid in achieving this goal. Only recently, RNAseq was exploited to refine the cleavage specificity of mycobacterial MazF, in a heterologous expression system in *E*. *coli* [53]. In contrast to the current understanding of canonical TA systems, in which the toxin is continuously inhibited by the antitoxin unless conditions become unfavorable, we observed activity of *S*. *aureus* MazF in the WT strain without shifting the toxin:antitoxin ratio to higher levels of free toxin. Intuitively, under standard laboratory conditions, the bulk of MazF toxin is expected to be complexed with MazE antitoxins. Studies in the past relied on overexpression of the toxin which most likely leads to unnatural abundances of proteins. For future work in *S*. *aureus*, it is therefore a promising approach not to overexpress the TA components but to rely on the natural activation of MazF to better resemble physiological conditions.

Interestingly, cleavage at UACAU sites was stimulated by heat shock, which indicates a link between MazF activity and the alternative σ^B^ factor. At this point it is unknown if this is due to an activation of only MazF, or transcriptional activation of the whole locus (including *mazF*) or another mechanism. The association of *mazEF* with the *rsbUVW-sigB* locus is found throughout the staphylococci (S3 Fig). This conserved synteny and the putatively vital role of the *mazEF* promoter in full σ^B^ activation [25] suggests a functional relationship between both loci. It is therefore probable, that MazF post-transcriptionally regulates the expression of other genes in concert with σ^B^. The enhanced sensitivity phenotype of the Δ*mazEF* strain towards the β-lactams penicillin and oxacillin is not due to a different growth rate compared to the WT (S7 Fig), instead it is putatively caused by one or more as yet unknown targets of MazF. These may be involved in cell wall synthesis or turnover, such as penicillin binding protein genes. Notably, *mazE* expression did not reinstate this phenotype, which underscores that the mechanism by which β-lactam sensitivity is mediated by the *mazEF* locus is unclear. Of note, the observed phenotype is not due to drug tolerance as observed with persister cells, because it was accompanied by altered MIC values observed with the WT vs. the deletion and/or the complementation mutant. Altered β-lactam-sensitivity can therefore be more interpreted as a specific regulatory property of the *mazEF* locus.

Our findings might be exploited in a clinical setting to fight *S*. *aureus* infections. It is conceivable that MazF-specific inhibitors increase β-lactam sensitivity of *S*. *aureus*. By contrast, considerations on the use of TA systems to fight bacterial infections so far focused on the activation of the toxin [54]. The suitability of TA systems as targets for antibacterial drugs must be thoroughly tested in the future. It is possible, that either activation or inhibition of the toxin leads to desired effects and this outcome may depend on the individual system. Whereas the biochemical and genetic characteristics of the staphylococcal *mazEF* system are increasingly better understood, it still remains enigmatic, whether this system plays a distinct physiological role and what it might be. We hope future research will help to shed light on this question.

## Supporting Information

S1 Fig Primer extension against the *spa* transcript, comparing HG003 with the Δ*mazEF* mutant. A strong cleavage signal at the first UACAU site (green) is visible in HG003 but absent in Δ*mazEF*. A VUUV’ site (blue) is not cut. See Fig 1 for more information.
(EPS)

S2 Fig Transcriptional starting point of the induced P_xyl/tet_ promoter, elucidated using a *gfp* primer on pRAB11 *gfp*. (EPS)

S3 Fig (A) Genetic organization of the *mazEF*-*sbUVW-sigB* locus in *S*. *aureus*. According to literature [25], transcription of the *mazEF* locus (dark blue arrows) is driven by the P_*mazEF*_ promoter and can either stop at a weak transcriptional terminator downstream of *mazEF* or continue to create a long transcript comprising the *mazEF* and *rsbUVW-sigB* locus (purple). Expression of *rsbU* is also controlled by promoter P_A_ and continues to the end of *sigB*. Promoter P_B_ is responsible for a shorter transcript containing *rsbVW* and *sigB*. Genes are denoted as filled arrows, promoters as angled arrows, transcriptional terminators as hairpin loops and transcripts by thin black lines. *hyp*. = hypothetical protein **(B) Synteny of the *mazEF-rsbUVW-sigB* locus and adjacent genes in other staphylococcal species.** Gene organization and genomic neighborhood of *mazEF-rsbUVW-sigB* is highly conserved in many staphylococcal species, indicating a functional link. Homologous regions are indicated by red boxing, flanking genes are colored light blue.
(EPS)

S4 Fig Protein A-sfGFP expression in *S*. *aureus* HG003 and Δ*mazEF*. **(A)-(C):** Growth at 37°C. The fluorescence of the overnight cultures (ONCs) was measured in the morning. The exponential phase cultures were inoculated from the 37°C ONCs to an OD_578_ of 0.07, incubated for 3 or 5 hours, respectively, at 37°C and then used for fluorescence measurements. **(D)-(E):** ONCs were grown at 37°C, but then re-inoculated to 42°C. Samples were compared using a paired two-tailed student’s t-test. Asterisks (*) indicate p-values < 0.05 when compared to HG003 WT and pound sign (#) p-values < 0.05 when compared to Δ*mazEF*.
(EPS)

S5 Fig RsbW-sfGFP expression in *S*. *aureus* HG003 and Δ*mazEF*. **(A)-(C):** Growth at 37°C. The fluorescence of the overnight cultures (ONCs) was measured in the morning after 2 hours of induction with ATc. The other cultures were inoculated from the ONCs to an OD_578_ of 0.07, induced after 1 hour, incubated for an additional 2 or 5 hours, respectively, and then used for fluorescence measurements. **(D)-(F):** ONCs were grown at 37°C but induced at 42°C. Exponential phase cultures were grown and induced at 42°C from 37°C ONCs. Samples were compared using a paired two-tailed student’s t-test. Asterisks (*) indicate p-values < 0.05 when compared to HG003 WT and pound sign (#) p-values < 0.05 when compared to Δ*mazEF*.
(EPS)

S6 Fig *S*. *aureus* Δ*mazEF* autolysis assay. HG003 (pRAB11-*gfp*), Δ*mazEF* (pRAB11-Pr-*mazEF*) and Δ*mazEF* (pRAB11-*gfp*) were subjected to 0.05% Triton X-100 and the optical density was measured over the course of five hours. The Δ*mazEF* deletion strain showed slightly higher autolysis rated than HG003 and the complemented Δ*mazEF* strains, intermediate lysis behavior.
(EPS)

S7 Fig Growth curves of different strains under non-selective conditions. **(A)** HG003 and Δ*mazEF*. **(B)** HG003 Δ*mazEF* with various *mazEF* complementation plasmids containing stop codons in either *mazE*, *mazF* or both genes.
(EPS)

S1 Table List of strains, plasmids and primers used in this study. (XLSX)
